# Blocking lncRNA *MALAT1*/miR-199a/ZHX1 Axis Inhibits Glioblastoma Proliferation and Progression

**DOI:** 10.1016/j.omtn.2019.09.005

**Published:** 2019-09-17

**Authors:** Keman Liao, Yingying Lin, Weizhen Gao, Zhipeng Xiao, Rogelio Medina, Pauline Dmitriev, Jing Cui, Zhengping Zhuang, Xiaochun Zhao, Yongming Qiu, Xiaohua Zhang, Jianwei Ge, Liemei Guo

**Affiliations:** 1 Department of Neurosurgery, Renji Hospital, School of Medicine, Shanghai Jiaotong University, No. 160, Pujian Road, District Pudong, Shanghai 200127, China; 2 Neuro-Oncology Branch, Center for Cancer Research, National Cancer Institute, NIH, Bethesda, MD 20892, USA; 3 Department of Neurosurgery, Barrow Neurological Institute, St. Joseph’s Hospital and Medical Center, 350 W. Thomas Road, Phoenix, AZ 85013, USA

**Keywords:** *MALAT1*, ZHX1, glioblastoma, competing endogenous RNA, long non-coding RNA

## Abstract

Zinc fingers and homeoboxes 1 (ZHX1) is a transcription repressor that has been implicated in the tumorigenesis and progression of diverse tumors. The functional role and regulating mechanism of ZHX1 has not been elucidated in glioblastoma (GBM). Previous reports have suggested that a large number of non-coding RNAs play a vital role in glioma initiation and progression. This study aimed to investigate the functional role and co-regulatory mechanisms of the metastasis-associated lung adenocarcinoma transcript-1 (*MALAT1*)/ microRNA-199a (miR-199a)/ZHX1 axis in GBM. We analyzed the expression of the *MALAT1*/miR-199a/ZHX1 axis and its correlation with patients’ overall survival using two different glioma gene-expression datasets. A series of *in vitro* and *in vivo* studies including dual luciferase reporter assay, fluorescence *in situ* hybridization (FISH), RNA immunoprecipitation, and pull-down experiments were completed to elucidate the biological significance of the *MALAT1*/miR-199a/ZHX1 axis in promoting glioma proliferation and progression. Elevated ZHX1 expression correlated with poor prognosis in GBM patients, and *in vitro* studies demonstrated that ZHX1 attenuated GBM cell apoptosis by downregulation of pro-apoptotic protein (Bax) and upregulation of anti-apoptotic protein (Bcl-2). Furthermore, knockdown of *MALAT1* inhibited GBM proliferation and progression *in vitro* and reduced tumor volume and prolonged survival in an orthotopic GBM murine model. Finally, we demonstrated that *MALAT1* promoted ZHX1 expression via acting as a competing endogenous RNA by sponging miR-199a. The *MALAT1*/miR-199a/ZHX1 axis promotes GBM cell proliferation and progression *in vitro* and *in vivo*, and its expression negatively correlates with GBM patient survival. Blocking the *MALAT1*/miR-199a/ZHX1 axis can serve as a novel therapeutic strategy for treating GBM.

## Introduction

Glioblastoma (GBM) is the most prevalent and aggressive primary malignant brain tumor with a median survival of 14.6 months.^1^ Many advances have been made in the effort to integrate diverse treatment strategies, including immunotherapy, stereotactic radiotherapy, and new chemotherapeutic agents; however, despite these efforts, patients with GBM still face a grim prognosis.^2^, ^3^, ^4^ Investigations into the molecular pathophysiological mechanisms driving GBM development and progression hold great promise for the identification of novel therapeutic targets and for guiding future treatments.

Zinc fingers and homeoboxes 1 (ZHX1) is a nuclear transcription repressor that interacts with the A subunit of nuclear factor-Y (NF-YA) and contains two C2H2-type zinc fingers and five homeobox DNA-binding domains.^5^ This protein has recently been implicated in the carcinogenesis of various cancers, including GBM.^6^, ^7^, ^8^ The mechanisms behind ZHX1’s potentially carcinogenic role have not been clearly elucidated, and previous reports suggest that ZHX1 may have both pro- and anti-carcinogenic functions among different cancer types. Kwon et al.^8^, ^9^ reported that ZHX1 expression levels positively correlated with increased proliferation, migration, and invasion of GBM and cholangiocarcinoma cells. However, other studies reported that ZHX1 suppressed proliferation of cancer cells. Ma et al.^10^ reported that ZHX1 was downregulated in gastric cancer tissues and that ZHX1 inhibited gastric cancer cell growth through inducing cell-cycle arrest and apoptosis. Wang et al.^11^ described that ZHX1 expression was reduced among cancer tissues from hepatocellular carcinoma patients, and overexpression of ZHX1 could inhibit the proliferation of hepatocellular carcinoma cells. Thus, the exact function of ZHX1 in carcinogenesis is not completely understood and its precise role in the pathogenesis of GBM remains to be elucidated.

Recently, a large number of non-coding RNAs (especially microRNAs [miRNAs] and long non-coding RNAs [lncRNAs]) have attracted increased attention due to their biological effects on GBM initiation and progression.^12^, ^13^, ^14^, ^15^, ^16^ miRNA-199a was found to be significantly downregulated in glioma tissues, and glioma patients with low miR-199a expression levels had decreased survival compared to patients with higher miR-199a levels.^17^, ^18^, ^19^ Coincidentally, miR-199a was identified to bind the ZHX1 3′ untranslated region (3′ UTR) to regulate ZHX1 expression, which contributed to carcinogenesis and progression in hepatic and gastric cancer cells.^20^, ^21^ Further investigation into miR-199a revealed its carcinogenic role in numerous other types of cancers, including prostate cancer,^22^ ovarian carcinoma,^23^ and osteosarcoma.^24^

Considering miR-199a’s role in the carcinogenesis of glioma and its potential regulatory effect on ZHX1, we conducted a bioinformatics analysis, which predicted a binding interaction between miR-199a and *MALAT1* (metastasis-associated lung adenocarcinoma transcript-1), a lncRNA. *MALAT1*, first discovered as a predictive factor for metastasis in early-stage, non-small cell lung cancer,^25^ was revealed to be one of the highly expressed lncRNAs in GBM tissues and corresponded to poor prognosis for GBM patients.^26^, ^27^, ^28^, ^29^ Additionally, recent studies have highlighted *MALAT1*’s potential role as a competing endogenous RNA (ceRNA), a class of miRNA that is capable of serving as a “molecular sponge” for smaller miRNAs, thereby modulating their downstream functions.^30^, ^31^, ^32^

In this study, we first revealed that ZHX1 is overexpressed in GBM tissues. Further study involving a series of *in vitro* and *in vivo* assays demonstrated that *MALAT1* could regulate ZHX1 expression by acting as a ceRNA against miR-199a in GBM proliferation and progression. These data provide novel insights into the role of ZHX1 and *MALAT1* in GBM progression and identify potential therapeutic targets in patients with GBM.

## Results

### ZHX1 Expression Is Elevated in Glioma Patients and Is Correlated with Poor Prognosis

**Figure 1:**
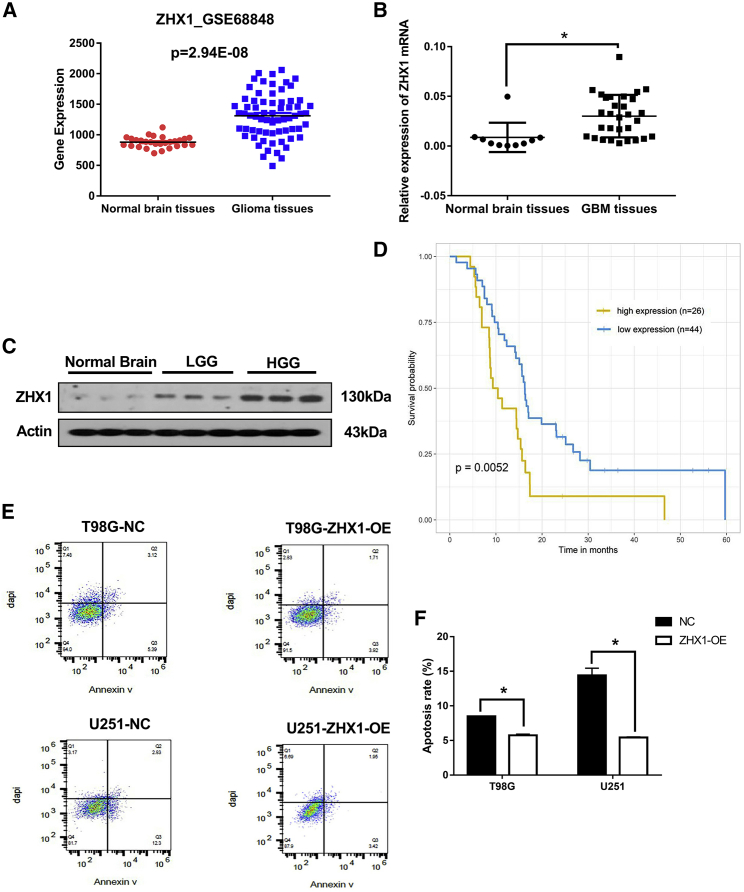
Elevated ZHX1 Expression Level in Glioma and Its Clinical Significance (A) The relative expression level of ZHX1 mRNA in GEO database (GEO: GSE68848). (B) The relative expression level of ZHX1 mRNA was analyzed by quanitative real-time PCR in 30 GBM tissues and 10 normal brain tissues collected from Renji Hospital. (C) Western blot analysis of ZHX1 in normal brain tissues, low-grade glioma (LGG, WHO grade II) tissues and high-grade glioma tissues (HGG, WHO grade IV). (D) Kaplan-Meier analysis for ZHX1 expression in GBM tissues of the GEO database (GEO: GSE7696). (E) Flow cytometry analysis of glioma cells (T98G and U251) with and without ZHX1-OE. (F) Quantification of apoptosis rate of glioma cells (T98G and U251) with and without ZHX1-OE. *p < 0.05.

Analysis of the tissue microarray data from the GEO database (GEO: GSE68848) demonstrated that the ZHX1 mRNA level in glioma tissue was significantly higher compared to normal brain tissues (Figure 1A). Analysis of the brain tissues from the Department of Neurosurgery, Renji Hospital, School of Medicine, Shanghai Jiaotong University similarly revealed that ZHX1 mRNA levels were significantly higher in GBM tissue (n = 30), compared to normal brain tissue (n = 10) (Figure 1B). Further, ZHX1 protein expression levels were significantly higher in high-grade glioma (WHO grade IV) and low-grade glioma (WHO grade II) tissues, compared to the normal brain tissue (Figure 1C). Data from the GEO database (GEO: GSE7696) was used to assess whether GBM patients survival correlated with ZHX1 expression level. Kaplan-Meier analysis revealed that GBM patients with high ZHX1 expression (30% upper percentile, n = 45) survived longer than the patients with low ZHX1 expression (30% lower percentile, n = 45) (Figure 1D; p = 0.0409).

### ZHX1 Attenuated GBM Cell Apoptosis and Was Accompanied by Upregulation of Bax and Downregulation of Bcl-2

**Figure 2:**
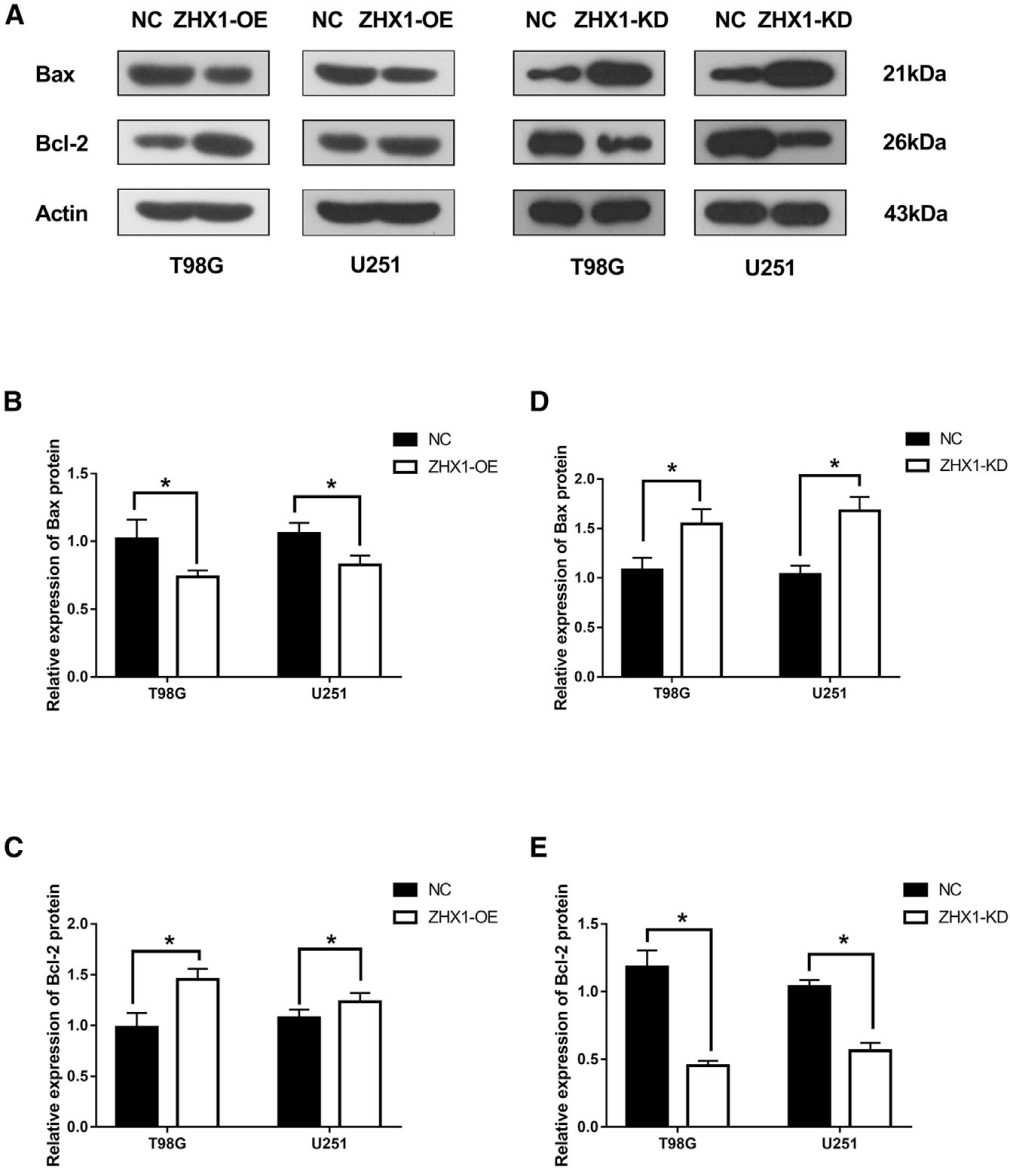
The Expressions of Apoptotic Regulator Proteins after ZHX1-OE or ZHX1-KD (A) Western blot analysis of pro-apoptotic protein (Bax) and anti-apoptotic protein (Bcl-2) in glioma cells (T98G and U251) with ZHX1-OE or ZHX1-KD. (B and C) Western blot densitometric quantification of Bax (B) and Bcl-2 (C) in glioma cells (T98G and U251) with ZHX1-OE. (D and E) Western blot densitometric quantification of Bax (D) and Bcl-2 (E) in glioma cells (T98G and U251) with ZHX1-KD. *p < 0.05.

Considering the inverse relationship between ZHX1 gene expression and patient survival, we investigated the role of ZHX1 in GBM cell apoptosis via flow cytometry. GBM (T98G and U251) cells with ZHX1 overexpression demonstrated a reduced apoptotic rate, compared to control cells (Figures 1E and 1F; p < 0.05). We also assessed the expression of apoptotic regulators, Bax and Bcl-2, and found that overexpression of ZHX1 in GBM (T98G and U251) cells was associated with decreased expression of Bax (pro-apoptotic protein) and increased expression of Bcl-2 (anti-apoptotic protein), whereas knockdown of ZHX1 produced the opposite effect (Figure 2; p < 0.01). These results suggest that ZHX1 attenuated GBM cell apoptosis by downregulation of Bax and upregulation of Bcl-2 directly or indirectly.

### *MALTA1* Level Is Elevated in GBM, and Knockdown of *MALAT1* Inhibited Cell Proliferation, Promoted Cell Apoptosis, and Decreased Invasion *In Vitro*

Because *MALAT1* is one of the few significantly upregulated lncRNAs in GBM,^26^, ^27^, ^28^, ^29^ we assessed *MALAT1* expression in brain tissue from the Department of Neurosurgery, Renji Hospital, School of Medicine, Shanghai Jiao Tong University. Results revealed that *MALAT1* expression was significantly higher in GBM tissue, compared to normal brain tissues (Figure S1A). Considering that the expression of *MALAT1* and ZHX1 demonstrated a similar trend in GBM tissues, a correlation analysis was completed. Results demonstrated that ZHX1 mRNA level was indeed positively correlated with *MALAT1* level (R^2^ = 0.3443, p < 0.01) (Figure S1B).

To investigate the role of *MALAT1* in regulating ZHX1 expression, we knocked down *MALAT1* in glioma cell lines with short hairpin RNAs (shRNAs). First, *MALAT1* expression was measured in 4 glioma cell lines (A172, T98G, U251, and U87); U251 and T98G cells demonstrated higher expression of *MALAT1* and were used in subsequent experiments (Figure S2A). Next, shRNAs against *MALAT1* were designed and assessed for knockdown efficacy; shRNA-1 demonstrated the best *MALAT1* knockdown efficacy and was used in subsequent experiments (Figure S2B).

**Figure 3:**
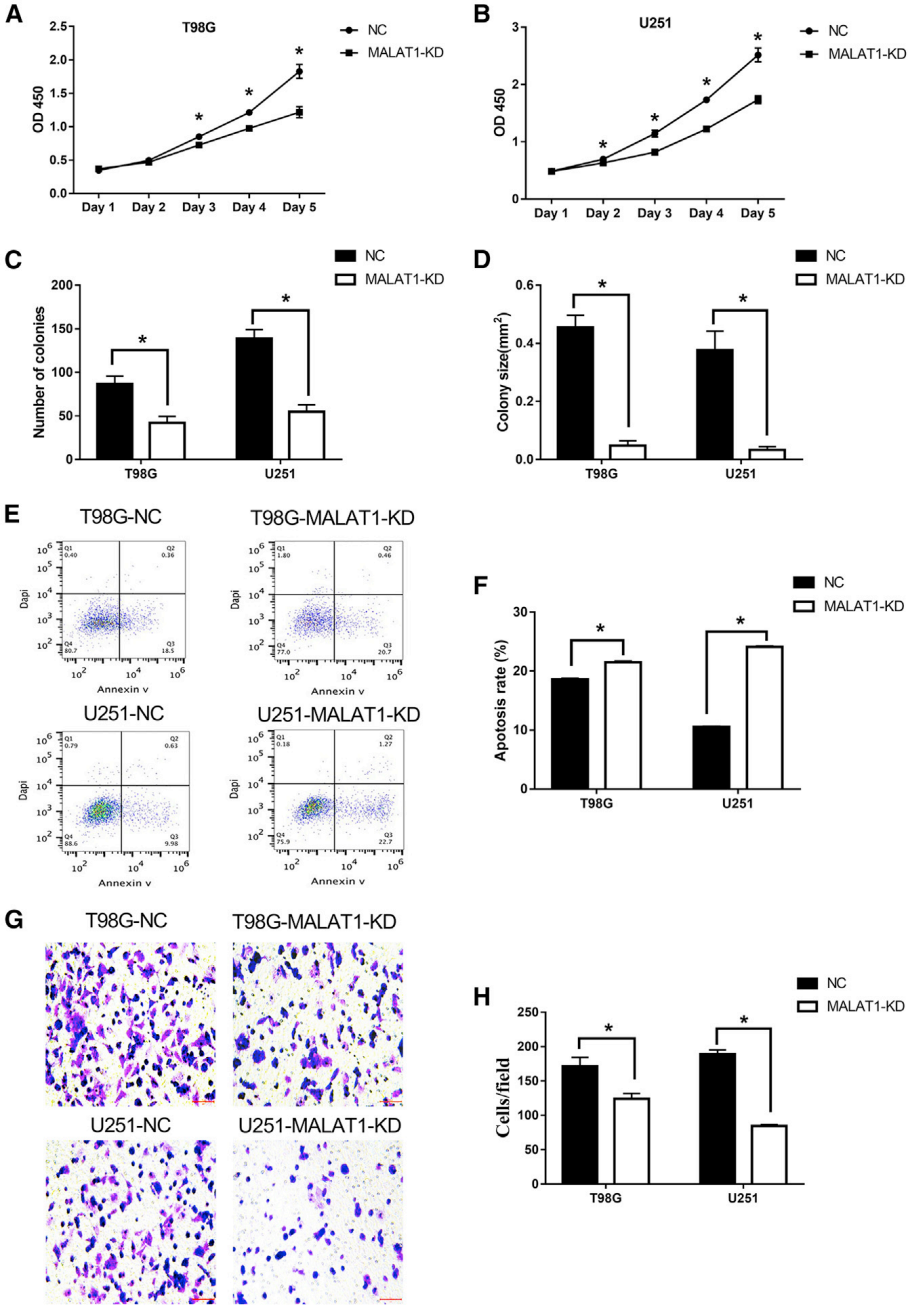
Knockdown of *MALAT1* Inhibited Cell Proliferation, Promoted Cell Apoptosis, and Decreased Invasion *In Vitro* (A and B) CCK-8 assays of T98G (A) and U251 (B) cells with and without *MALAT1*-KD. At the indicated time points, the number of cells per well was measured by the absorbance (450 nm). Colony assays of T98G and U251 cells with and without *MALAT1*-KD. (C and D) The number of colonies (C) and the mean colony area (mm^2^) (D) were determined using ImageJ analysis software (version 1.48). (E and F) Apoptosis analysis (E) and quantification (F) of T98G and U251 cells with and without *MALAT1*-KD. (G and H) Matrigel-Transwell assays (G) and quantification (H) of T98G and U251 cells with and without *MALAT1*-KD. *p < 0.05.

Knockdown of *MALAT1* demonstrated the following results: it inhibited T98G and U251 cell proliferation in the Cell Counting Kit-8 (CCK-8) assay (Figures 3A and 3B; p < 0.05), reduced the number of GBM colonies and colony sizes in the colony formation assay (Figure S3) (Figures 3C and 3D; p < 0.05), increased the apoptotic rates of T98G and U251 cells (Figures 3E and 3F; p < 0.01), and reduced the invasive ability of GBM cells in the Matrigel invasion assay (Figures 3G and 3H; p < 0.05). Collectively, these findings provide evidence that *MALAT1* contributes to glioma tumorigenesis and plays a crucial role in promoting its proliferation and progression.

### Knockdown of *MALAT1* Inhibited GBM Progression and ZHX1 Expression *In Vivo*

**Figure 4:**
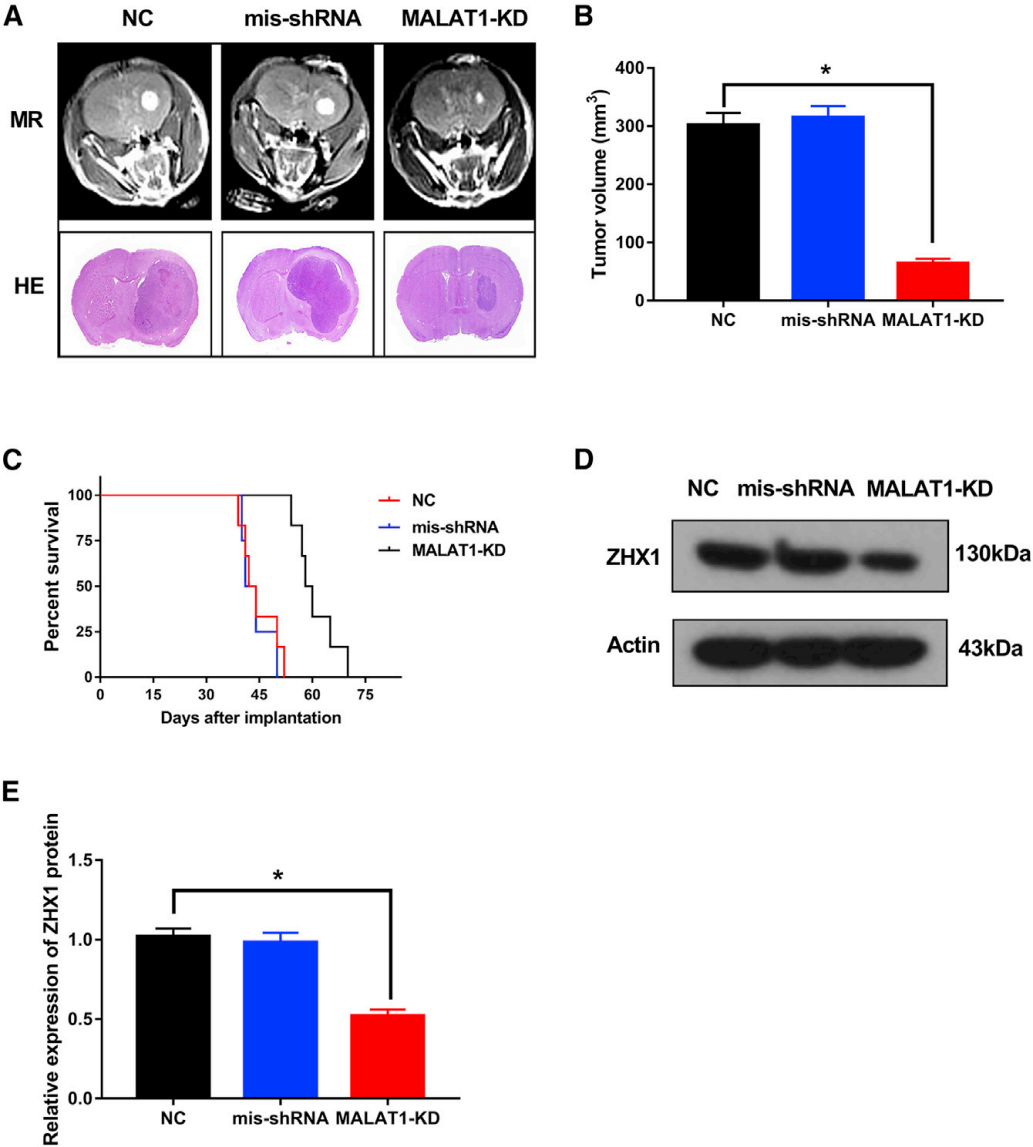
Knockdown of *MALAT1* Inhibited GBM Progression and ZHX1 Expression *In Vivo* (A) Representative axial MR images and H&E staining of xenograft GBM tumors in normal control, miR-199a inhibitor, and *MALAT1*-KD groups. (B) Tumor volume in normal control, miR-199a inhibitor, and *MALAT1*-KD groups. (C) Comparative survival of mice bearing normal control, miR-199a inhibitor, and *MALAT1*-KD. (D and E) Western blot analysis (D) and densitometric quantification (E) of ZHX1 in tumor tissues collected from nude mice injected with normal control, miR-199a inhibitor, and *MALAT1*-KD. *p < 0.05.

Considering that knockdown of *MALAT1* could inhibit ZHX1 expression and GBM progression *in vitro*, we assessed whether knockdown of *MALAT1* could similarly affect GBM progression *in vivo*. U251 cells transfected with *MALAT1*-shRNA (*MALAT1*-KD) or *MALAT1*-mis shRNA or saline (NC, control) were injected into the brain of severe combined immunodeficiency (SCID) mice. Subsequent mouse brain MRI and H&E staining (Figure 4A) revealed that tumor volume in the *MALAT1*-KD group was much smaller than the mis-shRNA group and the NC group (Figure 4B; p < 0.05). Survival time was also increased in the *MALAT1*-KD group compared to the mis-shRNA group and the NC group (Figure 4C; p < 0.05). Of note, ZHX1 protein expression was significantly lower in the *MALAT1*-KD group compared to the mis-shRNA group and the NC group (Figure 4D; p < 0.05). Taken together, these results suggest that *MALAT1* plays an important role in ZHX1 expression and promote GBM progression.

### *MALAT1* Promoted ZHX1 Expression via miR-199a in GBM Cells

**Figure 5:**
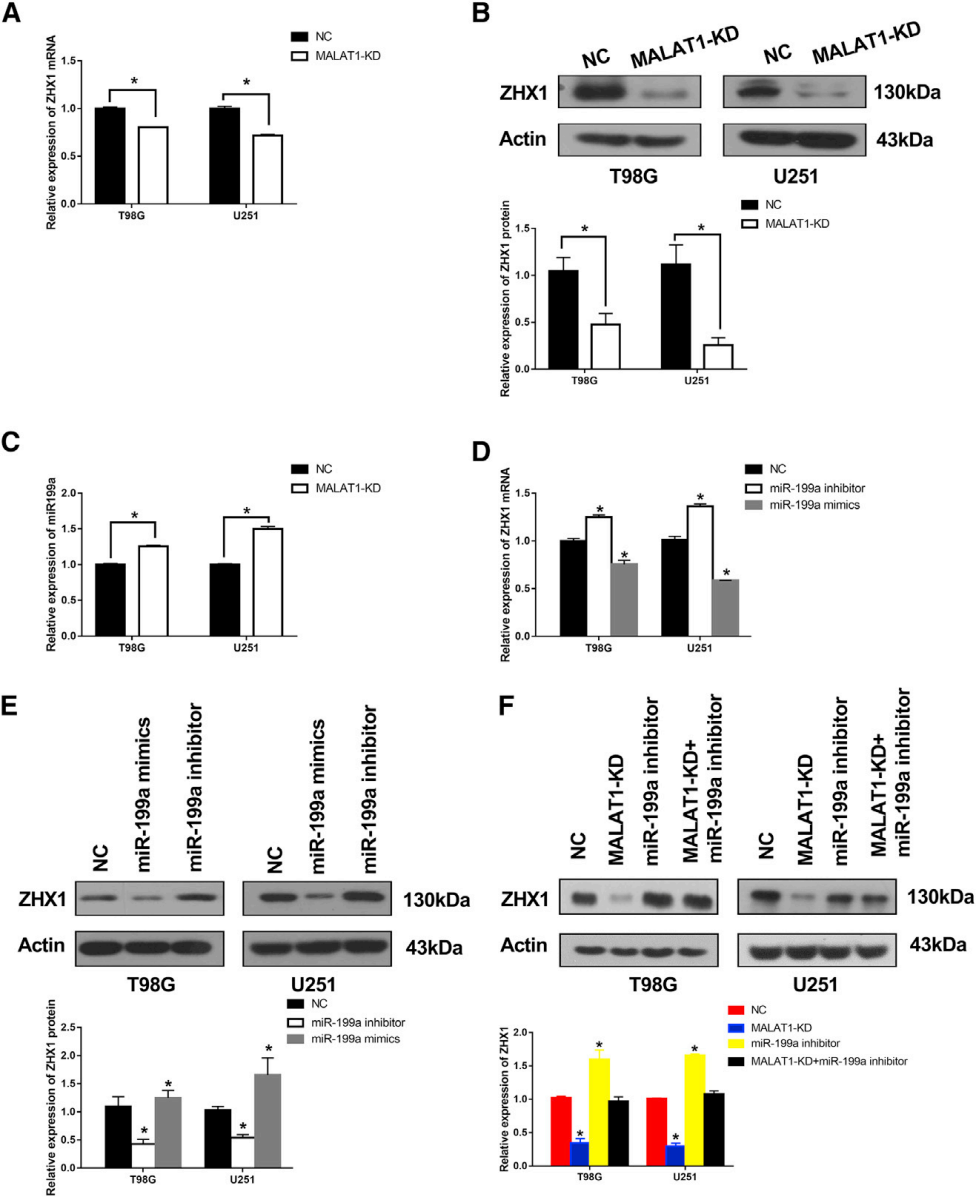
*MALAT1* Promoted ZHX1 Expression via miR-199a in GBM Cells (A and B) qRT-PCR and western blot analysis of ZHX1 mRNA (A) and protein (B) expression levels in T98G and U251 *MALAT1-*KD cells. (C) Knockdown of *MALAT1* increased miR-199a levels in T98G and U251 cells. (D and E) Ectopic miR-199a expression decreased ZHX1 mRNA (D) and protein (E) expression levels in T98G and U251 cells. (F) ZHX1 protein levels in T98G and U251 cells transfected with empty vector, or *MALAT1*-shRNA, or miR-199a inhibitor or both. *p < 0.05.

Knockdown of *MALAT1* in T98G and U251 cells significantly downregulated ZHX1 mRNA and protein levels (Figures 5A and 5B; p < 0.05). Hence, we investigated the expression level of miR-199a in brain tissues from the Department of Neurosurgery, Renji Hospital, School of Medicine, Shanghai Jiao Tong University. Results revealed that miR-199a levels were significantly lower in GBM tissues, compared to normal brain tissues (Figure S4A). A correlation analysis demonstrated that miR-199a level was negatively correlated with *MALAT1* level (Figure S4B; R^2^ = 0.252, p < 0.01). Additionally, knockdown of *MALAT1* significantly increased miR-199a level in T98G and U251 cells (Figure 5C; Figure S5; p < 0.05).

We also investigated the interaction between miR-199a and ZHX1 using miR-199a mimics and miR-199a inhibitors. Results of these experiments revealed that the miR-199a mimic reduced ZHX1 mRNA and protein levels in T98G and U251 cells, while the miR-199a inhibitor increased both mRNA and protein levels (Figures 5D and 5E; p < 0.05). Lastly, knockdown of *MALAT1* in T98G and U251 cells significantly decreased the expression of ZHX1 protein (Figure 5F; p < 0.05), however, this inhibitory effect was attenuated when cells were co-transfected with miR-19a inhibitor (Figure 5F). Taken together, these results suggest that *MALAT1* promotes ZHX1 expression via miR-199a in GBM cells.

### *MALAT1* Acts as a ceRNA against miR-199a, and ZHX1 Is a Direct Target of miR-199a in GBM Cells

**Figure 6:**
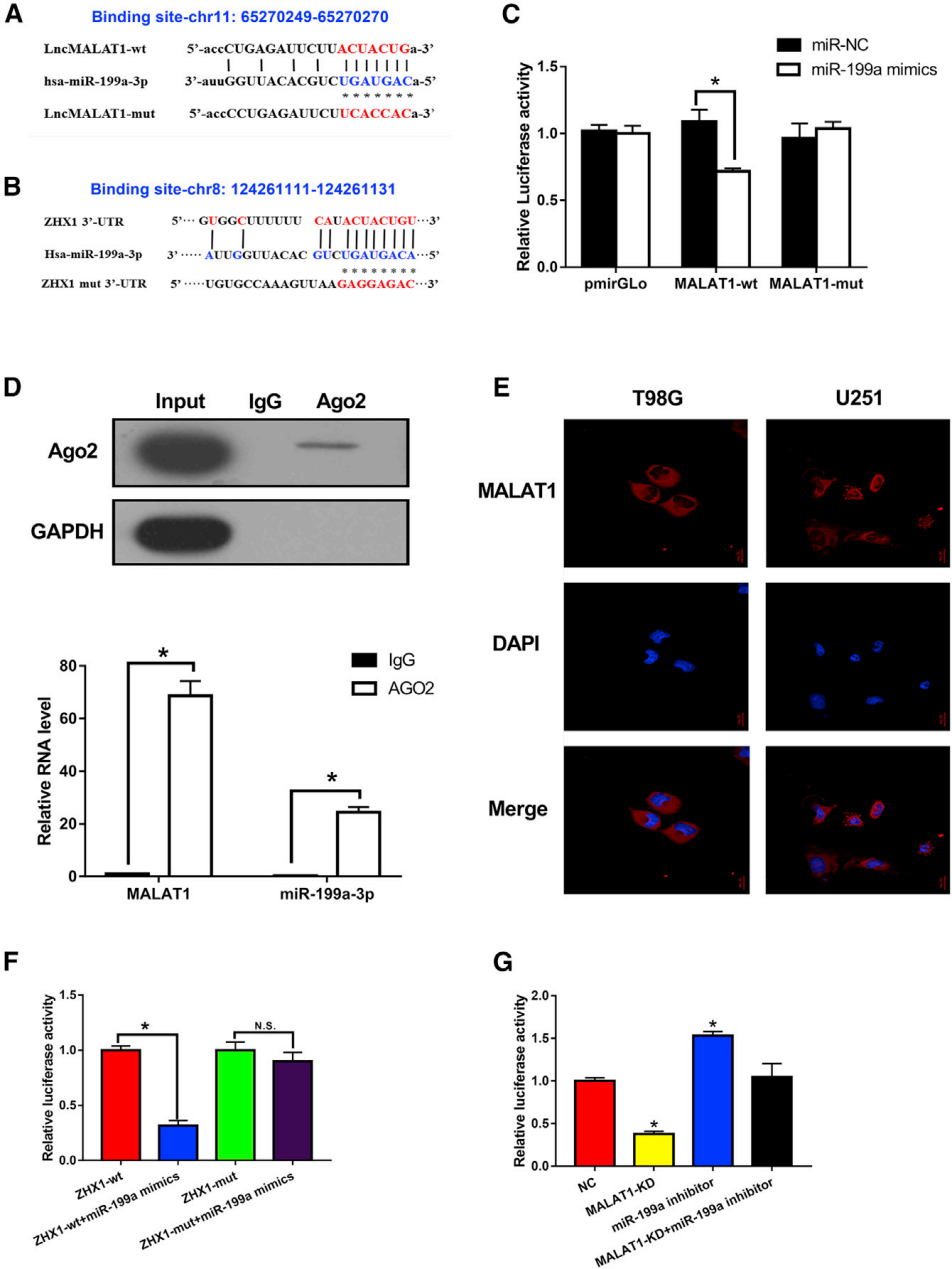
*MALAT1* Acted as a ceRNA against miR-199a, and ZHX1 Was a Target of miR-199a in GBM Cells (A) Schematic representation of the putative miR-199a binding sites in *MALAT1*. (B) Schematic representation of the putative miR-199a binding sites in ZHX1. (C) Dual-luciferase reporter assay in cells co-transfected with the WT or mutant reporter plasmid and miR-199a. (D) RIP assay using anti-Ago2, compared to anti-IgG in cells. (E) Subcellular fractionation and FISH staining confirmed the expression of *MALAT1* in cytoplasm. (F) Dual-luciferase reporter assay in ZXH1-WT and ZHX1-mut cells transfected with different vectors. (G) Luciferase reporter assay in HEK293T cells co-transfected with the Luc-ZHX1-3′ UTR and the indicated miRNAs. *p < 0.05.

To explore the molecular mechanisms underlying *MALAT1* regulation of ZHX1, we first screened the miRNA candidates that could bind to *MALAT1*. Starbase v2.0 was used to identify miR-199a that could potentially bind to *MALAT1*. The fragment that included binding sites between *MALAT1* and miR-199a, as predicted by bioinformatics analysis (Figure 6A), was cloned into a pmirGLO vector as the wild type (*MALAT1*-WT). A mutated vector (*MALAT1*-mut) was also generated by replacing the binding site with its complimentary sequence. Using miRanda software (http://www.microrna.org/microrna/home.do), we discovered that miR-199a could potentially bind to the 3′ UTR of ZHX1 mRNA. The predicted fragment, including binding sites, was cloned into the WT (ZHX1-WT) and mutated fragment (ZHX1-mut) (Figure 6B).

A dual-luciferase reporter assay was used to verify the binding of *MALAT1* and miR-199a; assay results revealed that the miR-199a mimic reduced the luciferase activity of *MALAT1*-WT, but not of *MALAT1*-mut (Figure 6C; p < 0.05). And then, a RNA-binding protein immunoprecipitation (RIP) assay was used to investigate whether *MALAT1* and miR-199a are involved in the formation of the RNA-induced silencing complex (RISC). RIP assay results revealed a significant enrichment of *MALAT1* and miR-199 using anti-Ago2 compared to anti-immunoglobulin G (IgG) (Figure 6D; p < 0.05). Furthermore, we examined the cellular localization of *MALAT1*. Subcellular fractionation analysis and FISH staining validated the cytoplasmic expression of *MALAT1* in T98G and U251 cells (Figure 6E). These results provide strong evidence that miR-199a binds to *MALAT1* in a RISC manner. Lastly, a dual-luciferase reporter assay was similarly used to verify the direct binding of ZHX1 mRNA and miR-199a; assay results revealed that miR-199a mimic reduced the luciferase activity of ZHX1-WT, but not of ZHX1-mut (Figure 6F; p < 0.05). These results suggest that miR-199a exerts a direct inhibitory effect on ZHX1 expression via binding to the 3′ UTR of ZHX1.

Lastly, to test the co-regulatory mechanism of the *MALAT1*-miR-199a-ZHX1 axis, luciferase reporter plasmids containing the 3′ UTR of ZHX1 were constructed. As expected, knockdown of *MALAT1* significantly decreased luciferase activity in HEK293T cells transfected with Luc-ZHX1-3′ UTR (Figure 6G; p < 0.05); conversely, luciferase activity could be rescued with miR-199a inhibitor.

### The *MALAT1*-miR-199a-ZHX1 Axis Promoted GBM Proliferation and Progression

**Figure 7:**
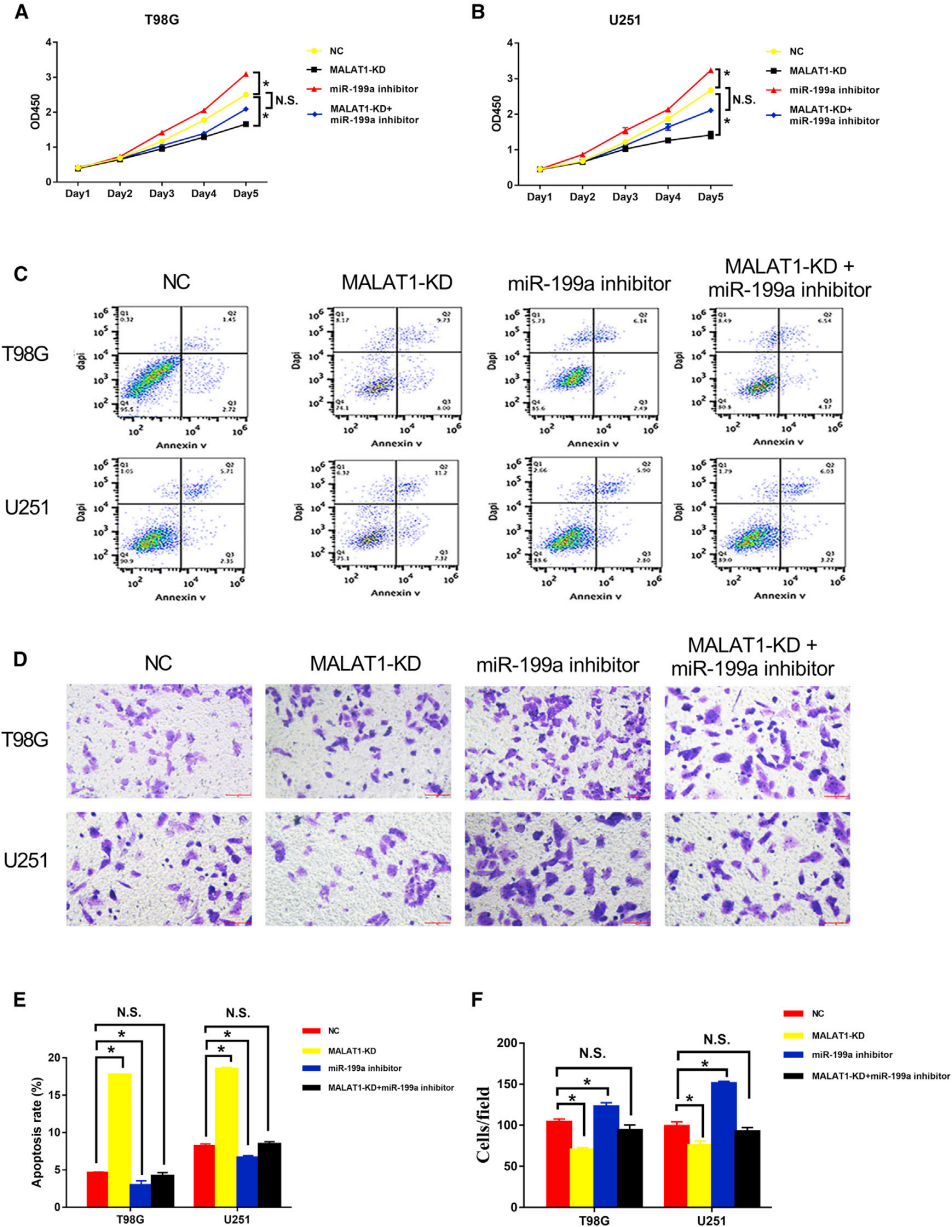
*MALAT1*-miR-199a-ZHX1 Axis Promoted GBM Proliferation and Progression T98G and U251 cells were transfected with empty vector, or *MALAT1*-shRNA, or miR-199a inhibitor or both for subsequent assays. (A) CCK-8 assays following transfection with different vectors. (B) Apoptosis assays following transfection with different vectors. (C) Matrigel-Transwell assays following transfection with different vectors. (D) Quantification of apoptosis rates following transfection with different vectors. (E) Quantification of transwell invasion cells following transfection with different vectors.

*In vitro* studies were conducted to investigate the biological effect of the *MALAT1*-miR-199a-ZHX1 axis in GBM cells. The results demonstrated that knockdown of *MALAT1* significantly inhibited cell proliferation (Figures 7A and 7B), promoted cell apoptosis (Figures 7C and 7E), and reduced cell invasion (Figures 7D and 7F) in T98G and U251 cells. Furthermore, the aforementioned tumor-promoting mechanisms were attenuated by miR-199a inhibitor. Taken together, these data indicate that the *MALAT1*-miR-199a-ZHX1 axis promotes GBM proliferation and progression.

## Discussion

ZHX1 is a novel transcription repressor localized in the nucleus and has been implicated in the carcinogenesis of various cancers.^6^, ^7^ However, the pathophysiological mechanism of ZHX1 during carcinogenesis is poorly characterized, especially in the context of glioma. In this study, we demonstrated that elevated ZHX1 expression was correlated with poor prognosis in GBM patients, confirming Kwon et al.^8^’s findings linking ZHX1 overexpression and reduced GBM patient survival. Further, *in vitro* studies revealed that ZHX1 overexpression resulted in a reduction in GBM cell apoptosis via inhibition of apoptotic protein Bax and promotion of anti-apoptotic protein Bcl-2. Furthermore, we demonstrated that knockdown of *MALAT1* inhibited GBM proliferation and progression *in vitro* and reduced the tumor volume and prolonged the overall survival in an orthotopic GBM murine model. Lastly, we demonstrated a novel regulatory mechanism between *MALAT1* and ZHX1, whereby *MALAT1* acts as a ceRNA against miR-199a. Blockage of the *MALAT1*/miR-199a/ZXH1 axis inhibited GBM proliferation and progression, underscoring its potential therapeutic role in the treatment of GBM.

By demonstrating that ZHX1 attenuated GBM cell apoptosis by downregulation of Bax and upregulation of Bcl-2, we suggest that ZHX1 plays a carcinogenic role in gliomagenesis. This result supported Kwon et al.^8^, ^9^’s findings that ZHX1 exerted a carcinogenic role in GBM and cholangiocarcinoma cells. However, our results stand in contrast to findings reported by Ma et al.^10^ that ZHX1 was downregulated in gastric cancer tissues and inhibited gastric cancer cell growth via induction of cell-cycle arrest and apoptosis. Similarly, our results also stand in contrast to findings reported by Wang et al.^11^ that demonstrated markedly reduced ZHX1 expression levels in human hepatocellular carcinoma (HCC) tissues and significantly reduced HCC cell proliferation with increased ZHX1 expression.

Presently, the mechanisms behind ZHX1’s pro or anti-carcinogenic role in different cancer types are still not fully elucidated. A possible explanation for ZHX1’s dualistic carcinogenic role may be explained by its potential involvement in various key signaling pathways. Kwon et al.^8^ reported that ZHX1’s carcinogenic role in GBM could be attributed to its regulation of TWIST and SNAI2. In a second study on cholangiocarcinoma, Kwon et al.^9^ demonstrated that ZHX1 exerted its carcinogenic role via its effect on early growth response protein 1 (EGR1), a multifunctional transcription factor with diverse regulator roles, including cell survival and apoptosis. In our study, we found that ZHX1 similarly exerted a regulator role on glioma cell apoptosis and promoted glioma cell survival via upregulation of Bcl-2 and downregulation of Bax.

Next, we demonstrated that *MALAT1* promoted GBM proliferation and progression *in vitro*, and knockdown of *MALAT1* reduced tumor volume and significantly prolonged survival in an orthotopic mouse model. These findings suggested that *MALAT1* plays an oncogenic role in glioma tumorigenesis, which was consistent with previous reports describing *MALAT1*’s carcinogenic role in various cancer types.^26^, ^27^, ^28^, ^29^ However, other studies have suggested *MALAT1* plays a tumor suppressive role in glioma.^33^, ^34^ A study by Han et al. demonstrated that knockdown of *MALAT1* promotes glioma invasion and proliferation via its downstream effects on extracellular signal-regulated kinase/mitogen-activated protein kinase (ERK/MAPK) signaling activity and expression of matrix metalloproteinase 2 (MMP2).^34^

The ceRNA hypothesis was first proposed by Salmena et al.^35^ in 2011; in the ceRNA gene interaction network, which includes lncRNAs, miRNAs, and mRNA, lncRNAs can act as endogenous molecular sponges that competitively bind miRNAs via shared miRNA response elements with reverse complementary binding seed regions to indirectly regulate mRNA expression levels. Notably, the correlated lncRNAs, miRNAs, and mRNAs in the ceRNA regulatory network mainly interact with each other in the cytoplasm.^36^

**Figure 8:**
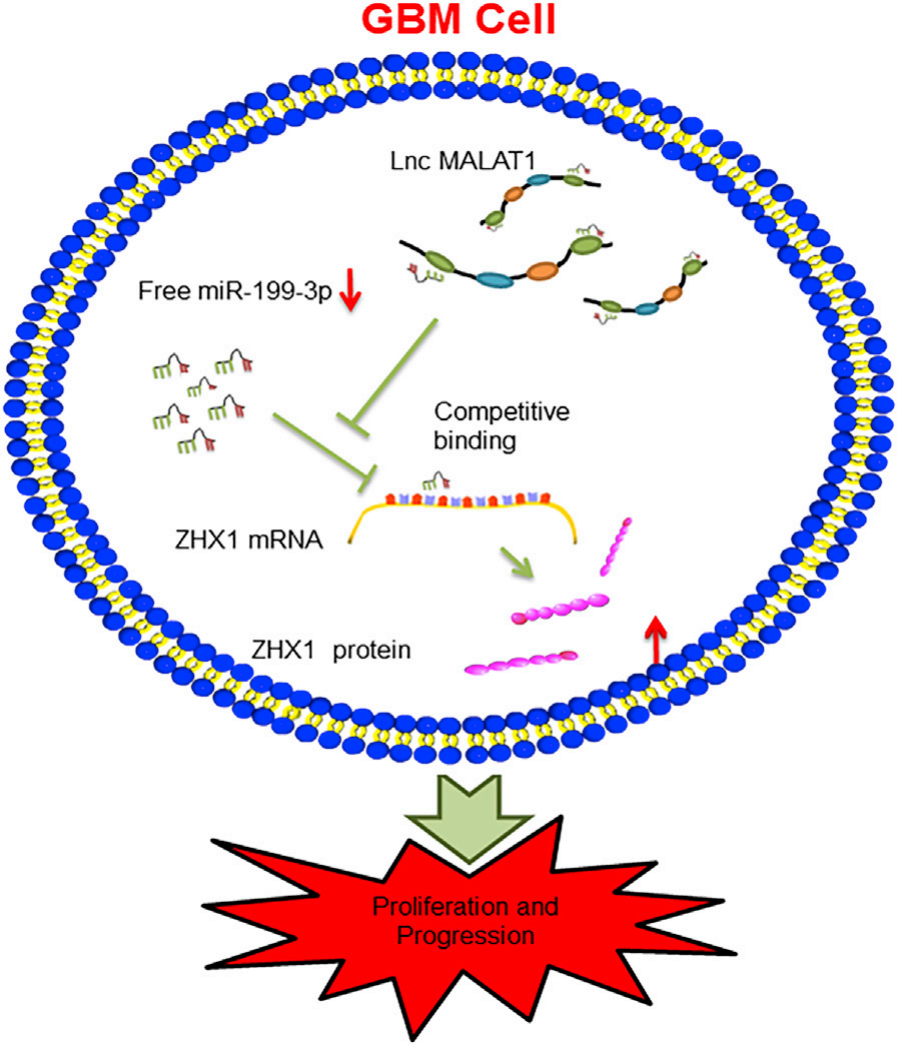
A Schematic Figure of the *MALAT1*/miR-199a/ZHX1 Axis A schematic figure describing the *MALAT1*/miR-199a/ZHX1 axis, showing that *MALAT1* regulates ZHX1 expression and glioma progression by acting as a ceRNA against miR-199a in GBM cells.

In the present study, we found that *MALAT1* and Id2 mRNA shared the same miR-199a binding sites. Furthermore, we affirmed that there was a reciprocal repression effect between *MALAT1* and miR-199a. More convincingly, we verified that only the WT *MALAT1* overexpression could affect ZHX1 expression; while the theoretical miR-199a binding sites in *MALAT1* were mutated, the facilitative effect of *MALAT1* on ZHX1 was dismissed. With dual luciferase reporter assay, RIP assay, and pull-down experiments, we demonstrated the dual competitive interaction of miR-199a with *MALAT1* and the 3′ UTR of ZHX1 mRNA. Lastly, with RNA FISH, we found that *MALAT1* expressed predominantly in the cytoplasm. Taken together, these outcomes certified that *MALAT1* regulated ZHX1 expression via sponging miR-199a via a ceRNA mechanism (Figure 8). In addition, accumulating evidence have implicated that *MALAT1* could serve as a ceRNA to sequester many other miRNAs, including miR-101, miR-129, miR-144, miR-211, and miR-203, in diverse oncological conditions.^27^, ^28^, ^30^, ^31^, ^32^

A potential explanation for discordant *MALAT1* study findings can lie in the fact that non-coding RNAs (including miRNAs and lncRNAs) have been found to dynamically interact with a network of downstream targets. The analysis of individual interactions within this miRNA regulatory network may yield findings to suggest that a specific lncRNA may play both a tumor suppressor and tumor promoter role in different cancer types, depending on the specific regulatory networks considered.^37^, ^38^ In our investigation, we specifically directed our focus on determining the role of *MALAT1* in the regulation of miR-199a and its downstream target, ZHX1. We accomplished this via a series of well-established *in vitro* and *in vivo* studies, which elucidated our mechanistic findings. Additionally, we correlated our experimental results with clinical and histopathological findings in GBM patients. Collectively, our comprehensive research methods and results support a carcinogenic role for *MALAT1* in the *MALAT1*-miR-199a-ZHX1 axis.

### Conclusions

In summary, our study demonstrated that elevated ZHX1 expression in GBM tissue was correlated with poor prognosis for GBM patients. Through a series of both *in vivo* and *in vitro* experiments, we demonstrated that *MALAT1* regulates ZHX1 expression by acting as a ceRNA against miR-199a in GBM cells. Lastly, we demonstrated that the *MALAT1*-miR-199a-ZHX1 axis promotes GBM cell proliferation and progression. Taken together, our results suggest that blockage of the *MALAT1*-miR-199a-ZHX1 axis could serve as a novel therapeutic target for GBM.

## Materials and Methods

### Human Tissues Collection and Gene-Expression Profiling Data

Glioma tissues (10 cases of astrocytoma [WHO grade II], and 30 cases of GBM) were obtained from patients in the Department of Neurosurgery, Renji Hospital, School of Medicine, Shanghai Jiaotong University in 2017. All patient specimens were placed in liquid nitrogen immediately after surgical removal. Their diagnoses were respectively confirmed by two pathologists and classified based on the 2007 World Health Organization (WHO) Classification of Tumors of the Central Nervous System. Additionally, 10 non-neoplastic brain tissues (regarded as normal control brain) from epileptogenic patients and their relevant clinical information were also collected at our department. All enrolled patients provided written informed consent, and the study protocol was approved by the Ethics Committee of Renji Hospital, School of Medicine, Shanghai Jiaotong University. All animal experiments were conducted in agreement with the Guide for the Care and Use of Laboratory Animals and were approved by the Committee on Animals handling of Shanghai Jiaotong University.

Glioma patient gene-expression profiling data (GSE68848 and GSE7696) was obtained from the NCBI GEO database. The GSE68848 database, which included 28 normal brain tissues and 67 glioma tissues, was used to investigate ZHX1 mRNA expression levels. The GSE7696 database, which included 4 normal brain tissues and 80 glioblastoma tissues, was used to investigate the relationship between overall survival and ZHX1 mRNA levels.

### Cell Lines and Cell Culture

The human glioma cell lines U87-MG, U251, T98G, and A172 (purchased from the Cell Bank of the Shanghai Branch of the Chinese Academy of Sciences) were maintained in DMEM supplemented with 10% FBS (Invitrogen) and 1% penicillin/streptomycin. Cells were cultured at 37°C in a humidified atmosphere of 5% CO_2_.

### Cell Transfection

ZHX1 was silenced by small interfering RNAs (siRNAs) targeting ZHX1 (5′- CUGACUUUUGAUGGUAGUU-3′, 5′-GAAAGUAAUGCAGGUAGUU-3′, 5′-CAGUUCAUCAUAACUCAGU-3′). Scramble siRNA (si-NC: 5′-GAUCCGCAAAAGAGCGAAA-3′) was negative control.

The overexpression and inhibition of miR-199a was realized by miR-199a mimics and miR-199a inhibitors, with NC mimics and NC inhibitors as controls (provided by QIAGEN). miR-199a mimic sense: 5′-ACAGTAGTCTGCACATTGGTTA-3′; antisense: 5′-TAACCAATGTGCAGACTACTGT-3′; miR-199a inhibitor: 5′-TAACCAATGTGCAGACTACTGT-3′. NC sense: 5′-UUCUCCGAACGUGUCACGUUU-3′; antisense: 5′-UUAAGAGGCUUGCACAGUGCA-3′.

The transfection of these plasmids into cells was carried out with Lipofectamine 3000 Reagent (Thermo Fisher Scientific, CA, USA) as required.

### Cell Proliferation Assay and Cell Apoptosis Assay

The CCK-8 assay was used to assess cell proliferation. Transfected cells (T98G and U251) were seeded on 96-well plates at a density of 2 × 10^3^/100 μL cells per well. CCK-8 (10 μL) was added into each well at 24-, 48-, 72-, 96-, and 120-h time points. After 2 h dark-room incubation at 37°C, each well was analyzed using a microplate reader (BioTek Elx800; BioTek Instruments, Winooski, VT, USA) at 450 nm.

To detect cell apoptosis, we double stained transfected cells (T98G and U251) with FITC-Annexin V and propidium iodide (PI) and subsequently analyzed them with flow cytometry (FACScan; BD Biosciences) equipped with Cell Quest software (BD Biosciences).

### Matrigel Transwell Assay

T98G and U251 cells were added to the upper chambers of Transwell assay inserts (Millipore, Billerica, MA, USA) with a Matrigel-coated membrane in 200 μL of DMEM without serum. The inserts were then placed into the bottom chambers in the 24-well plates, which contained DMEM with 10% FBS, and incubated for 24 h. The invading cells on the bottom surface were stained with 0.3% crystal violet. The invasion rate was quantified by counting the invading cells in at least three to five random fields.

### Colony Formation Assay

For colony formation assays, 6-well plates were seeded with transfected cells (T98G and U251) at a density of 500 cells/well and incubated for 10–14 days. The colonies were then fixed with 4% paraformaldehyde, stained with 0.1% crystal violet, and counted under a dissecting microscope.

### Retrovirus-Mediated ZHX1 Expression

For retrovirus-mediated ZHX1 expression: the ZHX1 gene was obtained from Invitrogen and was amplified using ZHX1 primers including XhoI and Miu I restriction sites. The amplified ZHX1 gene was cloned into the PHY-009 vector (purchased from Hanyin Shanghai, China; the main component of the vector is CMV-MCS-PGK-Blasticidin). The recombinant lentivirus and negative control (NC) lentivirus (Hanyin Shanghai, China) were prepared and titered to 10^9^ TU (transfection units)/mL. After 48 h, the overexpression or knockdown efficiency was confirmed by quantitative real-time PCR. Next, glioma cells (T98G and U251) were seeded in 6-well dishes at a density of 2 × 10^5^ cells per well, and after 24 h, infected with the same titer virus with 8 μg/mL polybrene. 72 h after the viral infection, the culture medium was replaced with medium containing 430 μg/mL blasticidin, and the glioma cells (T98G and U251) were cultured for at least 14 days. The blasticidin-resistant cells were cultured in medium containing 30 μg/mL blasticidin for 9 days and subsequently transferred to a blasticidin-free medium.

### *MALAT1* Knockdown with shRNAs

The *MALAT1* knockdown target sequence was: 5′-ACGGAAGTAATTCAAGATCAA-3′. The recombinant lentivirus and the NC lentivirus (Hanyin Shanghai, China) were prepared and titered to 10^9^ TU/mL. After 48 h, the knockdown efficiency was confirmed by quantitative real-time PCR. Next, glioma cells (T98G and U251) were seeded in 6-well dishes at a density of 2 × 10^5^ cells per well, and after 24 h, infected with the same titer virus with 8 μg/mL polybrene. 72 h after the viral infection, the culture medium was replaced with medium containing 4 μg/mL puromycin, and the glioma cells (T98G and U251) were cultured for at least 14 days. The puromycin-resistant cells were amplified in medium-containing 2 μg/mL puromycin for 9 days and subsequently transferred to a puromycin-free medium.

### Orthotopic Glioma Model and Treatment

We constructed an orthotopic murine model using U251 cells to verify the role of *MALAT1 in vivo.* Five-week-old male SCID mice (n = 21) were selected for this study. A total number of 2 × 10^6^ U251-sh-*MALAT1* or U251 -NC or U251-mis-shRNA cells in 5 μL DMEM were implanted into the corpus striatum of the mice as previously reported.^39^ Mice were monitored for tumor development by magnetic resonance imaging (MRI, 3-Tesla scanner, General Electric) 14 days after tumor implantation as previously reported.^39^ Mice were continuously observed until end points were reached in all animals.

### H&E Staining

The growth and invasion of implanted tumor were observed by routine H&E staining. All tissues were fixed in 10% formaldehyde for 24 h and then were dehydrated, permeated, wax-dipped, and embedded in paraffin. Tissues were subsequently cut into 3 μm sections. After staining with hematoxylin for 5 min and rinsed with running water for 5 min, tissue sections were soaked in hydrochloric acid solutions for 5 s, rinsed with running water for another 10 min, and then immersed in ammonia for 5 s. Tissue sections were then rinsed with running water for 10 min, stained with eosin solution for 30 s, rinsed with running water and immersed in distilled water briefly. Finally, the sections were rapidly dehydrated in graded ethanol (80%, 95%, and 100%), cleared in Xylene and mounted with neutral gum.

### RNA Extraction and Quantitative Real-Time PCR

Total RNA was extracted from tissue samples or cell lines using TRIzol reagent (Invitrogen, Carlsbad, CA, USA) according to the manufacturer’s instructions. The RNA concentration and quality were determined by UV spectrophotometry.

Quanitative real-time PCR was performed on a 7500 Real-Time PCR System (Applied Biosystems, Foster City, CA, USA) with Universal SYBR Green Master Mix (Roche, Shanghai, China). Reverse transcription of total RNA was performed using miScript II RT kit (QIAGEN [Cat No: 218160], Germany) and miScript SYBR Green PCR Kit (QIAGEN [Cat No: 218073], Germany) used for qPCR amplification of miR-199a level according to the provider’s instructions. MiRscript system is using added A-tail method to do reverse transcription. Mature miRNAs are polyadenylated by poly(A) polymerase and reverse transcribed into cDNA using oligo-dT primers. Polyadenylation and reverse transcription are performed in parallel in the same tube. The oligo-dT primers have a 3′ degenerate anchor and a universal tag sequence on the 5′ end, allowing amplification of mature miRNA in the real-time PCR step.

The following primers were used: *MALAT1* forward, 5′-TTGTAGACTGGAGAAGATAGG-3′ and reverse, 5′-ACTGAAGAGCATTGGAGAT-3′; and ZHX1 forward, 5′-GAAACAGATGATAGTGACACTTGG-3′ and reverse, 5′-TGATTCTCCTTCAACGTTTTAGGC-3′; and miR-199a primer 5′-ACAGUAGUCUGCACAUUGGUUA-3′; β-actin forward: 5′-CACCATTGGCAATGAGCGGTTC-3′; reverse: 5′-AGGTCTTTGCGGATGTCCACGT-3′; U6: forward 5′-CGCAAATTCGTGAAGCGTTC-3′. In addition, the sequences of miR-199a and U6 reverse universal primer were provided from QIAGEN kit, which were claimed confidential β-actin and U6 were used as an internal standard control for mRNA and miRNA detection, respectively. Each sample was replicated three times and data was analyzed by comparing Ct values.

### Western Blot

Western blot assay was performed to detect the expressions of ZHX1, Bax, and Bcl-2. Cells or tissues were lysed with radio immunoprecipitation assay buffer (RIPA, Beyotime, China) containing protease inhibitors (Roche, Complete Mini). The proteins (30 μg) were subjected to 8%–10% SDS-polyacrylamide gel electrophoresis and transferred onto Hybond ECL membranes (Amersham). The membranes were incubated for 1 h at room temperature (RT) in blocking buffer (5% skim milk in TBS-T) and then incubated with the appropriate antibodies (ZHX1 antibody [Cat. No: 13903-1-AP, Proteintech, 1:500]; Bax [Cat. No: 2772s, CST, 1:1000]; Bcl-2 [Cat. No: 2870s, CST, 1:1000]) and β-actin antibody from Abcam overnight at 4°C. After washing with TBS-T, the membranes were incubated with horseradish peroxidase-conjugated anti-rabbit or anti-mouse antibody (1:10,000 dilution; Sigma) for 2 h at room temperature. Detection was performed using western blot detection reagents (Odyssey).

### Luciferase Reporter Assay

HEK293T cells were seeded in 96-well plates and cultured to 50%–70% confluence before transfection. The cells were transfected with plasmid containing 3′ UTR of wild or mutant fragments from ZHX1 or *MALAT1* using Lipofectamine 3000 (Invitrogen, CA). After 48 h incubation, Promega Dual-Luciferase system was used to detect firefly and Renilla luciferase activities. Finally, the subtracted difference of firefly and Renilla luciferase activities were calculated as relative luciferase activity.

### RIP Assay

RIP assay was carried out using a Magna RIP Kit (Millipore) according to the manufacturer’s instructions. Whole cells were harvested and lysed in RIP lysis buffer with magnetic beads conjugated with human anti-Ago2 antibody (Cat. no: ab5072, Abcam) or IgG (Millipore, USA). The samples were incubated with Proteinase K and RNase inhibitors, and then immunoprecipitated RNA was isolated. Purified RNAs were extracted and analyzed by quanitative real-time PCR to demonstrate the presence of the binding targets. Each RIP assay was repeated three times.

### FISH

*MALAT1* probes were designed and synthesized by RiboBio (Guangzhou, China), and the probe sequences are available upon request. The probe signals were detected with a FISH Kit (RiboBio, Guangzhou, China) according to the manufacturer’s instructions. Briefly, GBM cells were fixed in 4% formalin for 15 min. After prehybridization in PBS, the cells were hybridized at 37°C for 30 min in hybridization solution. Then, cell nuclei underwent counterstaining by utilizing DAPI (4′,6-diamidino-2-phenylindole) staining (Beyotime, China). Images were captured using a fluorescence microscope (Leica, Germany).

### Statistical Analysis

Statistical analysis was performed on results from at least two independent replicates. Data were presented as means ± SD. The two-sided Student’s t test was applied to determine statistical significance between groups. Ordinary one-way ANOVA test was used for comparison between more than two groups. A p value less than 0.05 was considered statistically significant.

## Author Contributions

L.G. and X. Zhang designed the experiments, analyzed data, and wrote the article. K.L., Y.L., and W.G. performed the main experiments. Z.X., Y.Q., and J.G. helped with the experiments. R.M., P.D., J.C., Z.Z., and X. Zhao helped to analyze the data and revised the article. All authors read and approved the final manuscript.

## Conflicts of Interest

The authors declare no competing interests.

## Supplemental Information

Document S1. Supplemental Materials and Methods and Figures S1–S5

Document S2. Article plus Supplemental Information
