# Supplementary material for: Blocking lncRNA MALAT1/miR-199a/ZHX1 Axis Inhibits Glioblastoma Proliferation and Progression
Source: Mol Ther Nucleic Acids. 2019 Sep 17;18:388–99. doi: 10.1016/j.omtn.2019.09.005 (PMC6819876; doi:10.1016/j.omtn.2019.09.005)
Supplement: Document S1. Supplemental Materials and Methods and Figures S1–S5 [file mmc1.pdf]

OMTN, Volume *18*

Supplemental Information

Blocking lncRNA *MALAT1*/miR-199a/ZHX1 Axis Inhibits Glioblastoma Proliferation and Progression

Keman Liao, Yingying Lin, Weizhen Gao, Zhipeng Xiao, Rogelio Medina, Pauline Dmitriev, Jing Cui, Zhengping Zhuang, Xiaochun Zhao, Yongming Qiu, Xiaohua Zhang, Jianwei Ge, and Liemei Guo

Supplementary Methods

Northern Blotting

Approximately 30 µg of total RNAs were run on 10% polyacrylamide gel and transferred to a Hybond-N+ membrane (GE Healthcare, UK). The same membrane was hybridized with probes, stripped and reprobed with 5’-end, 32P-labeled oligonucleotides complementary to targeted sequences. The oligonucleotide sequences are as follows: for miR-199a detection, 5’-GAACAGGTAGTCTGAACACTGGG-3’; U6: 5’-ACGAATTTGCGTGTCATCCTTGCG-3’. Results were visualized through phosphorimaging (Typhoon).

Supplementary figures and figure legends

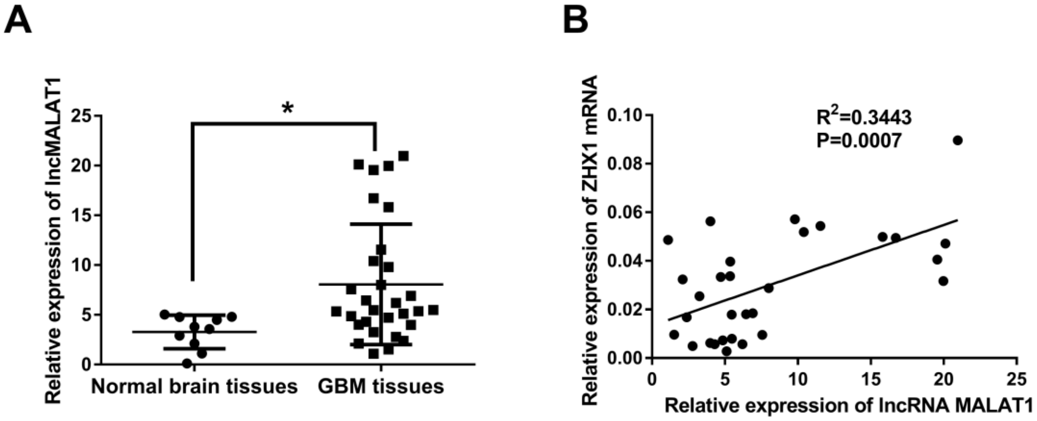

Figure S1. ZHX1 mRNA level was positively correlated with *MALAT1* level

(A) The relative expression level of *MALAT1* was analyzed by RT-qPCR in GBM tissue and normal control. (B) A correlation analysis demonstrated that ZHX1 mRNA level was positively correlated with *MALAT1* level (R^2=0.3443, $p<0.05$).

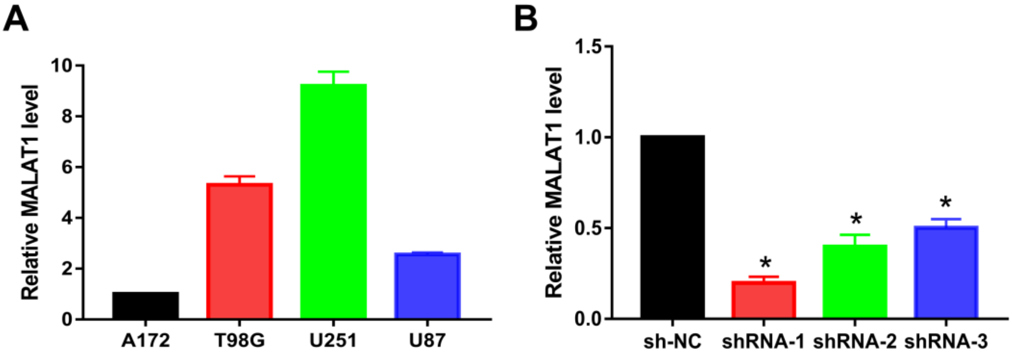

Figure S2. *MALAT1* expression levels in glioma cell lines and knockdown efficiency of *MALAT1* shRNAs

(A) *MALAT1* expression level was measured by RT-qPCR in 4 glioma cell lines (A172, T98G, U251, and U87). (B) shRNAs against *MALAT1* were designed and assessed by RT-qPCR for knockdown efficacy.

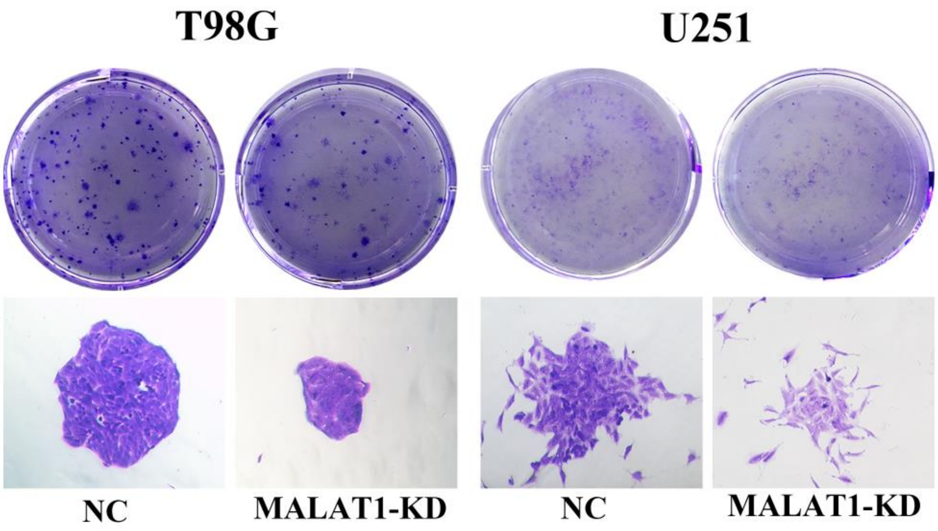

Figure S3. Knockdown of *MALAT1* in GBM cells reduced the number of GBM colonies and colony sizes

Knockdown of *MALAT1* in GBM cells (T98G and U251) reduced the number of GBM

colonies and colony sizes in the colony formation assay.

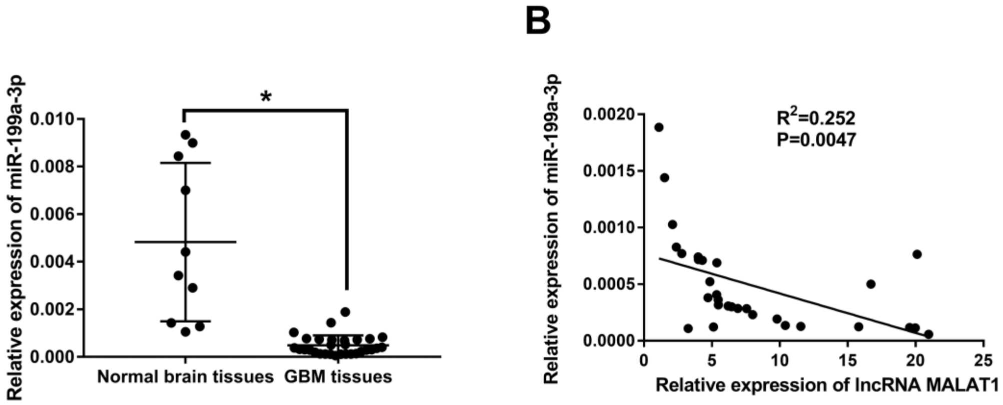

Figure S4. miR-199a level was negatively correlated with *MALAT1* level in GBM tissues

(A) The relative expression level of miR-199a was analyzed by RT-qPCR in GBM tissue and normal control. (B) Correlation analysis demonstrated that miR-199a level was negatively correlated with *MALAT1* level (R^2=0.252, p<0.01).

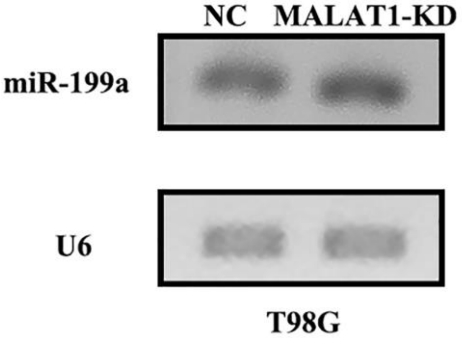

Figure S5. Northern blot analysis of miR-199a

Northern blot analysis of miR-199a in glioma cells with or without *MALAT1*-KD. U6 was used as a loading control.
